# Effect of remote nursing monitoring on overweight in women: clinical trial^*^

**DOI:** 10.1590/1518-8345.2651.3129

**Published:** 2019-03-18

**Authors:** Catia Suely Palmeira, Fernanda Carneiro Mussi, Carlos Antônio Souza de Teles Santos, Maria Lourdes Lima, Ana Marice Teixeira Ladeia, Lidia Cintia de Jesus Silva

**Affiliations:** 1 Universidade Federal da Bahia, Serviço Médico Universitário Rubens Brasil, Salvador, BA, Brazil.; 2 Universidade Federal da Bahia, Escola de Enfermagem, Salvador, BA, Brazil.; 3 Universidade Estadual de Feira de Santana, Departamento de Ciências Exatas, Feira de Santana, BA, Brazil.; 4 Escola Bahiana de Medicina e Saúde Pública, Salvador, BA, Brazil.

**Keywords:** Obesity, Monitoring, Telenursing, Health Education, Clinical Trial, Woman., Obesidade, Monitoramento, Telenfermagem, Educação em Saúde, Ensaio Clínico, Mulheres, Obesidad, Monitoreo, Teleenfermería, Educación en Salud, Ensayo Clínico, Mujeres

## Abstract

**Objective:**

to evaluate the effect of remote nursing monitoring on the improvement of anthropometric measurements of overweight women.

**Method:**

controlled, randomized clinical trial, carried out in a reference outpatient clinic for treatment of obesity. The baseline sample was composed of 101 women randomly assigned to two groups, 51 in the intervention group (IG) and 50 in the control group (CG). The IG received remote monitoring through telephone calls and conventional monitoring, and the CG received conventional monitoring. Women were assessed at the baseline and after three months of intervention. A paired t-test and analysis of covariance were used to evaluate intragroup differences in anthropometric measurements, and the statistical significance of 5% was adopted. Eighty one women completed the study.

**Results:**

in the intergroup comparison after the intervention, a reduction of 1.66 kg in the mean weight (p = 0.017) and of 0.66 kg/m^2^ in the mean BMI (p = 0.015) was found in the intervention group. There was a borderline statistically significant (p = 0.055) reduction of 2.5 cm in WC with in the intervention group.

**Conclusion:**

the remote monitoring was beneficial in reducing anthropometric measurements. RBR-3hzdgv.

## RESUMO

**Objetivo:**

avaliar o efeito do monitoramento remoto de enfermagem na melhora das medidas antropométricas de mulheres com excesso de peso.

**Método:**

ensaio clínico controlado, randomizado, realizado em ambulatório de referência em obesidade. Constituíram o *baseline* 101 mulheres, alocadas aleatoriamente em dois grupos, 51 no grupo de intervenção (GI) e 50 no grupo controle (GC). O GI recebeu o monitoramento remoto por telefone e acompanhamento convencional e o GC acompanhamento convencional. A avaliação das mulheres ocorreu no *baseline* e após três meses de intervenção. Para avaliar as diferenças das medidas antropométricas intragrupos, utilizou-se o teste t pareado e, intergrupos, a análise de covariância, adotando-se significância estatística de 5%. Completaram o estudo 81 mulheres.

**Resultados:**

na comparação intergrupos após a intervenção, verificou-se redução de 1,66 kg na média do peso (p = 0,017) e redução de 0,66 kg/m^2^ na média do IMC (p = 0,015) para o grupo intervenção. Houve redução de 2,5 cm na CC no grupo intervenção com diferença estatística *boderline* (p=0,055).

**Conclusão:**

o efeito do monitoramento remoto foi benéfico na redução das medidas antropométricas. RBR-3hzdgv.

## RESUMEN

**Objetivo:**

evaluar el efecto del monitoreo remoto de enfermería en la mejora de las medidas antropométricas de mujeres con sobrepeso.

**Método:**

ensayo clínico controlado, aleatorio, realizado en ambulatorio de referencia en obesidad. Constituyeron el *baseline* 110 mujeres, colocadas aleatoriamente en dos grupos, 51 en el grupo de intervención (GI) y 50 en el grupo control (GC). El GI recibió el monitoreo remoto por teléfono y el seguimiento convencional y el GC tuvo seguimiento convencional. La evaluación de las mujeres ocurrió en el *baseline* y después de tres meses de intervención. Para evaluar las diferencias de las medidas antropométricas intragrupos se utilizó el test t pareado y, intergrupos, el análisis de covariancia, adoptando significancia estadística del 5%. Completaron el estudio 81 mujeres.

**Resultados:**

en la comparación intergrupos después de la intervención se verificó reducción de 1,66 Kg en la media del peso (p=0,017) y reducción de 0,66 Kg m^2^ en la media del IMC (p=0,015) para el grupo intervención. Hubo reducción de 2,5 cm en la CC en el grupo intervención con diferencia estadística *boderline* (p=0,055).

**Conclusión:**

el efecto del monitoreo remoto fue benéfico en la reducción de las medidas antropométricas. RBR-3hzdgv.

## Introduction

Obesity is a complex chronic disease that has become a public health problem in many countries due to its high prevalence, causal relationship with many serious chronic diseases, negative effects on quality of life, and relevant economic consequences related to increased health care costs^1^ ^-^ ^2^. Overweight people are at higher risk of developing type 2 diabetes mellitus, cardiovascular disease, high blood pressure, certain types of cancer, osteoarthritis, and depression^3^.

Although there is a global obesity pandemic, the prevalence of overweight and obesity in men and women varies greatly within and between countries; there are more obese women than obese men, in general, especially in developing countries^4^. In Brazil, overweight has increased in all age groups, in both sexes, at all levels of income and schooling^5^.

The basic principle of fat accumulation that characterizes obesity and overweight is the energetic imbalance between calorie consumption and expenditure^4^. The causes of overweight are complex and multifactorial, including biological, environmental, social and psychological factors, making its control a challenge, especially the maintenance of long-term weight loss^6^ ^-^ ^7^. Although lifestyle interventions for weight loss are successful in the short term, weight regain is common^8^. Besides the few treatment options with proven efficacy for weight control, there is a lack of reference services with trained staff and an apparent lack of time and motivation of the clients that contribute to the problem^9^ ^-^ ^10^.

Although obesity is associated with genetic issues, the effect of genotype on its development is strongly influenced by other lifestyle factors. In modern societies, increased consumption of fat-rich and carbohydrate-rich foods with high energy density and low levels of physical activity has favored weight gain^3^.

Although there is no perfect evaluation for overweight and obesity, which may vary according to ethnic and genetic factors, the most commonly used body mass measurement has been height-adjusted weight^6^. A situation is classified as overweight when the body mass index (BMI) (weight in kg divided by the square of the height in meters) is above 24.9 kg/m^2(^ ^11^.

As excess visceral adipose tissue has been associated with metabolic syndrome and other chronic diseases, the assessment of visceral fat is indicated as an important marker. Waist measurement associated with BMI has been recommended for this evaluation^6^ ^,^ ^12^.

Research indicates that, in overweight people, small weight reductions, about 5 to 10%, may lead to improved control of major cardiovascular risk factors and prevent metabolic diseases^13^. Interventions that include behavioral changes and mainly address changes in eating habits and physical activity seem to aid in weight loss and long-term weight control^6^.

It is, therefore, essential to propose care to help people cope with the difficulties of weight loss and weight control and the problems arising from obesity. A study shows that nurses can implement programs for the evaluation and monitoring of chronic health problems and help people self-manage problems^14^. Health care should encompass a practical attitude mediated by the interaction of different sets of knowledge and should be based on a humanized relationship between the actors involved in situations that demand an integral therapeutic action, aiming at the best possible outcome^15^.

Remote monitoring is an innovative technology that can help people who experience in chronic health conditions, resulting in a greater sense of empowerment, better management of disease, and adherence to treatment. However, little is known about its benefits in the management of weight control. Researches that used this technology were mostly performed with people with other chronic health problems such as chronic obstructive pulmonary disease, congestive heart failure and diabetes mellitus^16^ ^-^ ^18^.

In this context, the present study aimed to evaluate the effect of remote nursing monitoring on the improvement of anthropometric measurements of overweight women.

## Method

This is a randomized controlled trial conducted at an outpatient reference service for obesity in the city of Salvador, Bahia, Brazil.

The project was approved by the Research Ethics Committee, CAAE: 43665115.6.0000.5531, and by the Brazilian Registry of Clinical Trials (RBR-3hzdgv). The study complied with Resolution 466/12 of the National Health Council of Brazil and followed the recommendations of the Consolidated Standards of Reporting Trials (CONSORT)^19^ for the randomization, follow-up and analysis of the data. All participants signed the Informed Consent Form.

Of the 317 women enrolled in the service, 117 met the inclusion criteria as they had BMI ≥ 25 kg/m^2^, were over 18 year old and younger than 60, and had attended at least one consultation in the last 12 months. We excluded women with cognitive difficulties and severe psychiatric disorders who were unable to respond to the questionnaires, women using weight loss drugs, who had undergone bariatric surgery, or who did not have a telephone device. The choice for carrying out the study with women was due to the fact that they represented 91% of the people enrolled in the service.

Women that met the inclusion and exclusion criteria (117) were invited to participate in the research and specific dates were scheduled for collecting the initial data, with about 20 participants per day. Of these, 101 participated and were part of the baseline sample. Before the collection, a playful workshop was held with the objective of raising expectations of guidance and knowledge about the difficulties faced to control weight; the workshop lasted one hour. The workshop and the data collection were carried out at the study site and had the participation of previously trained undergraduate nursing students and researchers.

**Figure 1:**
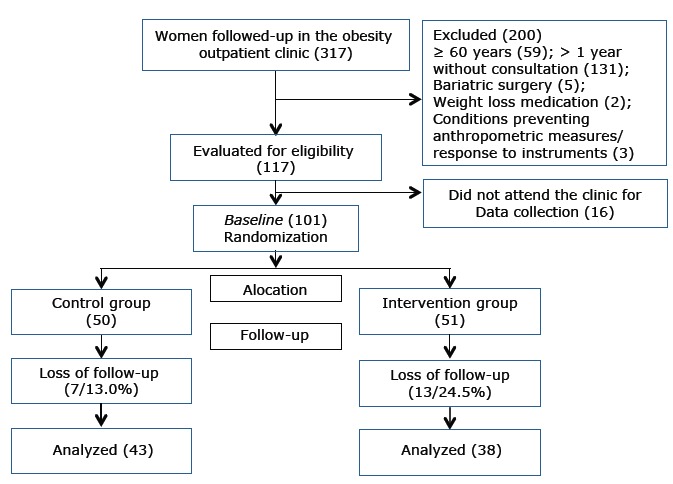
Flowchart of allocation of the study population

After the initial data collection with the baseline group of 101 women, they were randomized in blocks. The proposal established 51 women allocated to group A (intervention group) and 50 to group B (control group) according to the age group because age is a variable that can influence the response to treatment. Thus, the randomization was organized according to the age, which consisted in two groups: group 1: < 50 years, and group 2: ≥ 50 years. The group 1 had 51 women; 26 were randomly assigned to the intervention group A and 25 to the control group B. As for the second age group, the same procedure was applied. Figure 1 shows the research flowchart.

While the control group only attended routine follow-up consultations, the intervention group received remote monitoring consisting of telephone calls in addition to these consultations. As for the randomization process, the characteristics of the population were analyzed to ensure comparability between groups.

Data collection took place from July 2016 to March 2017. A form with closed questions was prepared for collection of sociodemographic data containing the variables of interest such as age, race/color, schooling, marital status, performance of paid activities, monthly family income, comorbidities, smoking, and alcohol consumption.

Physical activity was assessed by the validated long version of the International Physical Activity Questionnaire (IPAQ)^20^, with questions related to the frequency, duration and intensity of physical activities performed for more than ten continuous minutes during the last week covering the four domains of physical activity such as work, displacement, domestic activities and leisure. The IPAQ allows the classification of individuals as very active, active, insufficiently active and sedentary.

The food intake was evaluated by an instrument adapted from Vigitel^21^, with closed questions about the daily consumption of beans, vegetables, legumes, fruits, meats, soft drinks, cakes, pies and sweets, as well as the food preparation.

Anthropometric data were measured in private practices. The weight, in kilograms, was measured in a digital scale with the participant barefoot, wearing a minimum of clothes and without props. Height (in meters) was measured using a portable stadiometer (to an accuracy of ± 0.5 cm) with the participant standing, with the head and back leaning on the ruler of the stadiometer, below the horizontal rod, keeping the arms stretched and aligned with the body, with the shoulders, shoulder blades, buttocks, and heels leaning against the wall and feet resting on the floor. These two variables (weight and height) were used to calculate body mass index (BMI), whose formula is the ratio between the weight in kilograms and the square of the height in meters (BMI = weight/height^2^). BMI was classified according to WHO: overweight (BMI = 25 to 29.9 kg/m^2^), obesity I (BMI = 30 and 34.9 kg/m^2^), obesity II (35 and 39.9 kg/m^2^) and obesity III (≥ 40 kg/m^2^)^11^.

The waist circumference (WC) was measured at the midpoint between the last rib and the anterior superior iliac crest on the right axillary line, using a flexible and inelastic metric tape to an accuracy of 0.1 cm. The classification of WC in women followed the International Diabetes Federation (IDF) parameter: normal (< 80 cm) and increased (≥ 80 cm)^6^.

The remote monitoring lasted three months, included weekly telephone contact following a previously established weekly thematic protocol prepared with basis on the information from guides of scientific societies^6^ and consultation to specialists. The monitoring aimed to provide new guidelines, reinforce correct content, review inaccurate information, motivate continuity of treatment, and clarify doubts about overweight and control measures. The topics addressed were related to the disease, cause and complications of overweight, and control measures through the adoption of healthy eating habits and the practice of physical activity. The guidelines on eating habits addressed the importance of healthy eating, types of food, how to prepare food, and proper fractionation of meals.

As for the practice of physical activity, the information was mainly about its benefits, when and how to perform it, and the harm caused by physical inactivity. Guidelines on proper hydration and risk of diets and weight loss products without indication and supervision of a health professional were also included. Specific content related to women was allowed to be addressed, as well as the expression of doubts and comments. There was flexibility in the sequence of exposition of the content if need was perceived, asking the women to report if they were understanding the information on the topics presented. We sought to use language that is easy to understand, objective and attractive. To guide the team during the calls, a Telephone Call Guide was developed to be followed in each call before the implementation of the Weekly Thematic Guide Script.

The intervention and control groups were reassessed at the end of the intervention.

Categorical variables were analyzed through absolute and relative frequencies and continuous variables through measures of central tendency (mean) and variability (standard deviation). The paired t-test was used to analyze the intragroup differences in weight, after checking the normality of the data with the Shapiro-Wilk test. The weight between the intervention and control groups after the intervention was compared by analysis of covariance (ANCOVA), adjusted by the initial weight. Statistical significance was indicated by p values < 0.05.

The power of the sample was estimated based on the mean of the difference of the weight and respective SD, before and after RM, of the CG and IG, considering n1 = 50 (CG) and n2 = 51 (IG) and alpha of 0.05, reaching the power of 80.8%. The data were entered into the Statistical Package for Social Science (IBM SPSS version 18.0) and transported to STATA version 12.0 for analyses.

## Results

The sociodemographic characteristics of the participants of the intervention and control groups are shown in Table 1. The majority were women aged 50-59 (47.5%), mean age 47.8 (SD 9.0), of black/brown self-declared race/color (93.1%), complete and incomplete secondary education (62.4%), without a partner (51.5%), with paid work activity (54.5%), and a family income less than or equal to three minimum wages (87.1%), and average monthly income of 2.0 (SD 1.2) minimum wages. There was no statistically significant difference between the groups (CG and IG), making the sample homogeneous.

**Table 1:** Sociodemographic characteristics of overweight women at the baseline and according to the control and intervention groups. Salvador, BA, Brazil, 2017

**Variable**	**Total** **(n=101)**	**Control group (n=50)**	**Intervention group (n=51)**	Valor de p*
Age (years) ˘ **(SD** ^**†**^ **)**	**47.8 (9.0)**	**47.5 (8.8)**	**48.1(9.4)**	**0.775** ^**‡**^
**Age group (n/%)**				**0.482** ^**§**^
**< 50 years**	**53 (52.5%)**	**28 (56.0%)**	**25 (49.0%)**	
**≥50 years**	**48 (47.5%)**	**22 (44.0%)**	**26 (51.0%)**	
**Race/color (n/a)**				**0.715** ^**§**^
**Black/brown**	**94 (93.1%)**	**46 (92.0)**	**48 (94.1%)**	
**White**	**7(6.9%)**	**4 (8.0%)**	**3 (5.9%)**	
**Schooling (n/%)**				**0.514** ^**§**^
**Complete elementary education**	**30 (29.7%)**	**13 (26.0%)**	**17 (33.3%)**	
**Complete or incomplete high school**	**63 (62.4%)**	**34 (68.0%)**	**29 (56.9%)**	
**Complete or incomplete higher education**	**8 (7.9)**	**3(6.0%)**	**5 (9.8%)**	
**Marital status (n/%)**				**0.617** ^**‡**^
**Without partner**	**52 (51.5%)**	**27 (54.0%)**	**25 (49.0)**	
**Married/with a partner**	**49 (48.5%)**	**23 (46.0%)**	**26 (51.0)**	
**Paid work activity (n/%)**				**0.427** ^**‡**^
**Yes**	**55 (54.5%)**	**25 (50.0%)**	**30 (58.8)**	
**No**	**46 (45.5%)**	**25 (50.0%)**	**21 (41.2)**	
**Monthly family income** ^**||**^ **(n/%)**				**0.554** ^**‡**^
**≤ 3 SM** ^**||**^	**88 (87.1%)**	**45 (90.0%)**	**43 (84.3%)**	
**> 3 SM** ^**||**^	**13 (12.9%)**	**5 (10.0%)**	**8 (15.7%)**	

*p-value; ^†^SD - Standard Deviation; ^‡^Chi-square; §Fisher’s exact test; ^||^Minimum wage on 11/01/2017: R$ 937.00

Table 2 shows the description of the lifestyle variables and anthropometric measures of the participants. Although the majority of women presented a level of global physical activity considered active/very active (71.3%) and they were active/very active at home (51.5%), 82.2% were insufficiently active/sedentary at work, 76.2% in displacement and 85.2% in leisure time. The percentage of smoking was 4.0% and of alcohol use was 35.6%. It was verified that a greater proportion of the women consumed legumes (45.0%), vegetables (54.0%), and fruits (62.3%) with a frequency of five days or more in the week. A lower proportion consumed salad on five or more days in the week (47.0%) and had five or more daily meals (34.0%).

Regarding the anthropometric data, the control group and intervention group were homogeneous. A higher proportion of women were observed with obesity (84.5%) and all of them had increased WC (≥ 80 cm). The mean weight of the sample was 91.6 kg (SD = 15.5), the minimum was 61.6 kg and the maximum was 128.9 kg. The median was 90.7 kg.

**Table 2:** Level of physical activity, smoking, alcohol consumption, eating habits and anthropometric characteristics of overweight women in the baseline and according to the group they were assigned, control group and the intervention group. Salvador, BA, Brazil, 2017.

**Variables**	**Total** **(n=101)**	**Control group (n=50)**	**Intervention group (n=51)**	p-value*
**Level of physical activity**				
**Active/very active**	**72 (71.3%)**	**33 (45.8%)**	**39 (54.2%)**	**0.245** ^**†**^
**Inactive/sedentary**	**29 (28.7%)**	**17 (68.6%)**	**12 (41.4%)**	
**Smoking**	**4 (4.0%)**	**1 (25.0%)**	**3 (75%)**	**0.369** ^**†**^
**Consumption of alcohol**	**36 (35.6%)**	**18 (50.0%)**	**18 (50.0)%**	**0.553** ^**†**^
**Food consumption**				
**Legumes ≥ 5 days/week**	**45 (45.0%)**	**23 (51.1%)**	**22 (48.9%)**	**0.702** ^**†**^
**Vegetables ≥ 5 days/week**	**54 (54.0%)**	**26 (48.1%)**	**28 (51.9%)**	**0.345** ^**†**^
**Salad ≥ 5 days/week**	**47 (47.0%)**	**23 (48.9%)**	**24 (51.1%)**	**0.347** ^**†**^
**Fruits ≥ 5 days/week**	**63 (62.3%)**	**29(46.0%)**	**34 (54.0%)**	**0.456** ^**†**^
**Pies/cakes ≥ 3 days/week**	**9 (9.1%)**	**5 (55.6%)**	**4 (44.4%)**	**0.473** ^**‡**^
**Number of daily meals ≥ 5**	**34 (34.0%)**	**17 (50.0%)**	**17 (50.0%)**	**0.710** ^**†**^
**Preparing of cooked baked grilled foods**	**93 (93.9%)**	**44 (47.3%)**	**49 (52.7%)**	**0.112** ^**†**^
**Anthropometric measurements**				
**Weight (kg)** ˘ **(SD** ^**§**^ **)**	**91.6 (15.5)**	**93.7 (16.9)**	**89.5 (13.9)**	**0.132** ^**||**^
**Body mass index (n/%)**				
**Overweight**	**15 (14.9%)**	**7 (46.7%)**	**8 (53.3%)**	**0.235** ^**‡**^
**Obesity grade I**	**31 (30.7%)**	**11 (35.5%)**	**20 (64.55)**	
**Obesity grade II**	**23 (22.8%)**	**14 (60.9%)**	**9 (39.1%)**	
**Obesity grade III**	**32 (31.0%)**	**18 (56.3%)**	**14 (43.7%)**	
**Waist circumference (n/%)**				
**≥ 80 y <88 (cm)**	**5 (5.0%)**	**4 (80.0%)**	**1 (20.0%)**	**0.169** ^**‡**^
**≥ 88 (cm)**	**95 (95.%)**	**46 (48.4%)**	**49 (51.6%)**	

*p-value; ^†^Chi-square; ^‡^Fisher’s exact test; ^§^SD - Standard Deviation; ^||^t-test

Data on the intragroup comparison of the anthropometric measurements before and after the remote monitoring is presented in Table 3. When comparing the intragroup variation of weight before and after the intervention, an increase was observed in the control group (p = 0.041) and a decrease in the intervention group, but without statistical significance (p = 0.146).

There was an increase in average BMI in the control group with borderline statistical significance (p = 0.052) and there was no change in the intervention group (p = 0.144). No statistically significant changes were observed in waist circumference in the intragroup comparison before and after the intervention.

**Table 3:** Intragroup comparisons of anthropometric measurements before and after remote monitoring. Salvador, BA, Brazil, 2017

**Anthropometric measurements**	**Control group**			**Intervention group**		
	**Before n=50**	**After n=43**	p-value*	**Before n=51**	**After n=38**	p-value*
**Weight (mean/SD** ^**†**^ **)**	**93.8 (17.3)**	**94.7 (17.7)**	**0.041**	**88.8 (13.1)**	**88.0 (13.4)**	**0.146**
**BMI** ^**‡**^ **(mean/SD** ^**†**^ **)**	**37.5 (6.1)**	**37.9 (6.1)**	**0.052**	**34.9 (5.2)**	**34.7 (5.8)**	**0.144**
**WC** ^**§**^ **(mean/SD** ^**†**^ **)**	**106.7 (12.9)**	**108.4 (12.7)**	**0.107**	**104.2 (9.8)**	**103.6(10.0)**	**0.510**

*p-value obtained in the paired t-test; ^†^SD - Standard deviation; ^‡^BMI - Body mass index; ^§^WC - Waist circumference

Table 4 shows the results of the intergroup comparison after the intervention. There was a reduction of 1.66 kg in the mean weight (p = 0.017) and a reduction of 0.66 kg/m2 in the mean BMI (p = 0.015) of the intervention group. There was a reduction of 2.5 cm in WC in the intervention group with borderline statistical difference (p = 0.055) (Table 4). The model explained 96% of weight (R^2^ 0.966) and BMI (R^2^ 0.962) reduction and 77% of WC reduction (R^2^ 0.774).

**Table 4:** Effect of monitoring anthropometric measurements, comparing the control group and the intervention group. Salvador, BA, Brazil, 2017

**Anthropometric measurements**	**Coefficient**	SE*	**p-value** ^**†**^
**Weight (kg)**	**-1.66**	**0.68**	**0.017**
**BMI** ^**‡**^ **(kg/m²)**	**-0.66**	**0.26**	**0.015**
**WC** ^**§**^ **(cm)**	**-2.50**	**1.28**	**0.055**

*SE - Standard error; ^†^p-value obtained by the Analysis of Covariance (ANCOVA) adjusted for initial weight; ^‡^BMI - Body Mass Index; ^§^WC - Waist circumference

## Discussion

This randomized controlled trial evaluated the effect of remote nursing monitoring of overweight women and confirmed the benefits in terms of improved anthropometric measurements. The study provides evidence for the use of this therapeutic tool in health and nursing care.

Women who were the target of the remote monitoring were characterized predominantly by the low level of schooling and low income, in line with the literature. This indicates that excess weight is greater among people with lower socioeconomic level^22^. The relationship between weight accumulation and social conditions is explained by the fact that low-income people face more barriers to accessing healthy foods and practicing physical activity necessary for weight control^9^. The conditions of social inequality require sensitive approaches of the health team, in order to understand the difficulties of each person and adapt the guidelines accordingly.

Women participating in this study were predominantly black and the productive age range, but almost half did not have paid work. Most had domestic work as main occupation, which resulted in the classification of their level of activity as active or very active at home. Despite of this classification, a low percentage performed physical activity in the free time, in the displacement and in the professional working environment.

Physical inactivity is more prevalent among poorer women and generally combined with inadequate diet^23^, a profile similar to the population in this study. This situation is worrying because regular physical activity for at least 150 minutes per week is one of the pillars of weight reduction treatment^24^. Lack of time, poor access to affordable facilities, and limited space available for physical activity at home can explain the lack of physical activity during leisure time in people with more financial constraints^9^.

Still with respect to lifestyle, the major problems were related to the eating pattern, because smoking and alcohol consumption were not prevalent. The recommended frequency of consumption of legumes, vegetables, salads and fruits was not observed, the frequency was rather unsatisfactory, but higher than those found in women in Salvador (32.3%)^5^. A higher intake of these foods is recommended in view of their higher fiber, vitamins, antioxidants, minerals and unsaturated fats content, and lower glycemic load, salt and trans fat, which allows weight loss and weight maintenance^25^.

Regarding the number of meals per day, the frequency of women who did the fractionation of the diet, which helps in decreasing the desire to eat, was low. This type of strategy can also contribute to lower cholesterol levels, maintenance of blood glucose levels, control of appetite, and control of proper weight^26^.

The percentage of women with obesity was higher than that of overweight and all were with increased waist circumference, thus representing a population group with a high risk of morbidity and mortality. These parameters are strongly associated with a higher prevalence of type 2 diabetes mellitus and cardiovascular diseases^27^. Increased visceral adipose tissue is associated with a number of metabolic abnormalities, including reduced glucose tolerance and insulin sensitivity and adverse lipid profiles^28^.

Data from the present study showed that remote nursing monitoring was effective because the anthropometric measurements of the women in the intervention group presented a statistically significant reduction compared to the control group. Even small reduction of weight, BMI and waist circumference resulting from educational approaches should be seen as a positive thing if compared to weight reduction programs using medications where a reduction of 1% or more of body weight per month, reaching at least 5% in three to six months, is considered effective^6^.

Although a growing number of studies has investigated the use of information and communication technology for weight loss, the technical approaches used and the time of intervention differ considerably between them making it difficult to compare their findings with the present study. However, it is noteworthy that the use of these technologies also achieved favorable results in terms of weight loss^29^. Only one educational intervention study used face-to-face meetings and telemonitoring technology. It was a clinical trial that assessed the adherence of 170 adults to the weight loss program and found that the group that received the intensive education intervention presented a reduction of BMI of 1.0 kg/m^2^ on average compared to the less intensive intervention group^30^.

The management of overweight people should not be limited to the goal of great weight reduction and its maintenance over time, but should also contribute to the valorization of incorporation of healthy habits and improvement of clinical conditions. In this sense, remote monitoring represents an effective therapeutic tool for health education and self-care incentive. As for obesity, this strategy should be considered as an additional option to conventional treatment^31^.

The effect of the monitoring mediated through telephone, the strong point of the present study, confirms this method as effective to approach users in their homes, mainly as an educational strategy, due to flexibility of schedules, optimization of time and resources and capacity to reach large numbers of users who face difficulties involving geographic and financial barriers to access the health service^17^ ^,^ ^32^.

In chronic health conditions, such as heart failure, acute myocardial infarction, and chronic respiratory and renal diseases, telemonitoring has proved to have beneficial effects. These benefits generally refer to the empowerment of users to play an active role in their own health care, influencing their attitudes and behaviors, and improving clinical conditions^33^.

The results of this research may contribute to an innovative perspective for health professionals, especially nursing professionals, who work with overweight people. The difficulties to lose weight and maintain a healthy lifestyle are already confirmed in the literature and nurses can act at the forefront of care for prevention and control of excess weight using remote monitoring associated with face-to-face meetings with users in the services where they work, especially in primary health care.

During the development of this study it was possible to perceive other benefits of remote monitoring. They include the regular contact with the participants of the intervention group, which favored the exchange of information on necessary measures for weight control, clarification of doubts, incentive to adhere to the therapy, stimulus to maintain or adhere to healthy eating habits and practice of physical activity. It also helped to enhance the feeling of being cared for by nurses. Telemonitoring represented a follow-up approach that helped users to manage excess weight and to obtain more favorable anthropometric indicators.

The limitations of the present study are related to the time of the intervention, considered short for a more beneficial effect. A longer follow-up period may more effectively reflect the effects of this type of intervention. Therefore, we suggest the evaluation of the effect of remote nursing monitoring of overweight women through a longer follow-up period.

## Conclusions

The results showed that the remote monitoring of weight had a beneficial effect of reducing the anthropometric measures of women in the intervention group when compared to the control group.

## References

[1] Hruby A, Hu FB (2015). The Epidemiology of Obesity A Big Picture. Pharmacol Econ.

[2] Bahia L, Coutinho ESF, Barufaldi LA, Abreu GA, Malhão TA, Souza CPR (2012). The costs of overweight and obesity BMC Public Health. Pharmacol Econ.

[3] Mitchell NS, Catenacci VA, Wyatt HR, Hill JO (2011). Obesity overview of an epidemic. Psychiatr Clin North Am.

[4] Kanter R, Caballero B (2012). Global Gender Disparities in Obesity A Review. Adv Nutr.

[5] Malta DC, Andrade SC, Claro RM, Bernal RTI, Monteiro CA (2014). Trends in prevalence of overweight and obesity in adults in 26 Brazilian state capitals and the Federal District from 2006 to 2012. Rev Bras Epidemiol.

[6] Associação Brasileira para o Estudo da Obesidade e da Síndrome Metabólica (2016). Diretrizes Brasileiras de Obesidade 2016 / ABESO - Associação Brasileira para o Estudo da Obesidade e da Síndrome Metabólica.

[7] Wanderley EM, Ferreira VA (2010). Obesity: a plural perspective. Ciênc Saúde Coletiva.

[8] DerSarkissian M, Bhak RH, Huang J, Buchs S, Vekeman F, Smolarz BG (2017). Maintenance of weight loss or stability in subjects with obesity a retrospective longitudinal analysis of a real-world population. Curr Med Res Opinion.

[9] Burgess E, Hassmén P, Pumpa KL (2017). Determinants of adherence to lifestyle intervention in adults with obesity a systematic review. Clin Obes.

[10] Teixeira FV, Pais-Ribeiro JL, Maia ARPC (2012). Beliefs and practices of healthcare providers regarding obesity: a systematic review. Rev Assoc Med Bras.

[11] World Health Organization (2004). Obesity: preventing and managing the global epidemic: report of a WHO consultation.

[12] Burton RF (2010). Waist circumference as an indicator of adiposity and the relevance of body height. Med Hypotheses.

[13] Brown JD, Buscemi J, Milsom V, Malcolm R, O'Neil PM (2016). Effects on cardiovascular risk factors of weight losses limited to 5-10 % Translation Behav. Med.

[14] Turner A, Anderson JK, Wallace LM, Bourne C (2015). An evaluation of a self-management program for patients with long-term conditions. Patient Educ Counsel.

[15] Silva JLL, Machado EA, Costas FS, Sousa JL, Taveira RP, Carolindo FM (2013). Relationship between health-disease process and cross-cultural care contributions to nursing care. Rev Pesqui Cuidado Fundam.

[16] Souza-Junior VD, Mendes IA, Mazzo A, Godoy S (2016). Application of telenursing in nursing practice an integrative literature review. Appl Nurs Res.

[17] Furuya RK, Mata LR, Veras VS, Appoloni AH, Dantas RA, Silveira RC (2013). Telephone Follow-Up for Patients After Myocardial Revascularization A Systematic Review. AJN.

[18] Lachtermacher AP, Tocantins FR (2013). Information and communication technology and the prevention of diseases - literature review Rev Pesq Cuid. Fundam.

[19] Gewandtera JS, Eisenachb J, Grossd RA, Jensenf MP, Keefeg FJ, Leei DA (2018). Checklist for the preparation and review of pain clinical trial publications a pain-specific supplement to CONSORT. PAIN Reports,.

[20] Matsudo SM, Araújo TL, Matsudo VKR, Andrade DR, Andrade EL, Oliveira LC (2001). International physical activity questionnaire (IPAQ) study of validity and reliability in Brazil. Rev Bras Ativ Saude.

[21] Moura SA, Bezerra IN, Cunha DB, Sichieri R (2011). Evaluation of food intake markers in the Brazilian surveillance system for chronic diseases - VIGITEL (2007-2009). Rev Bras Epidemiol.

[22] Dinsa G, Goryakin Y, Fumagalli E, Suhrcke M (2012). Obesity and socioeconomic status in developing countries a systematic review. Obesity Rev.

[23] Sá-Silva SP, Yokoo EM, Salles-Costa R (2013). Gender-specific demographic factors and lifestyle habits related to physical inactivity. Rev Nutr.

[24] Dias IB, Montenegro RA, Monteiro WD (2014). Physical exercises as a strategy to prevent and to treat obesity physiological and methodological aspects. Rev HUPE (Rio de Janeiro).

[25] Mozaffarian D (2016). Dietary and Policy Priorities for Cardiovascular Disease, Diabetes, and Obesity A Comprehensive Review. Circulation.

[26] Pereira LM, Vieira ALS, Santos PMHLC (2014). Women's meal frequency and nutritional and health profiles. Rev Nutr.

[27] Sangrós FJ, Torrecilla J, Giráldez-García C, Carrillo L, Mancera J, Mur T (2017). Association of General and Abdominal Obesity With Hypertension, Dyslipidemia and Prediabetes in the PREDAPS Study. Rev Esp Cardiol.

[28] Castro AVB, Kolka CM, Kim SP, Bergman RN (2014). Obesity, insulin resistance and comorbidities ? Mechanisms of association. Arq Bras Endocrinol Metab.

[29] Burke LE, Wang J, Sevick MA (2011). Self-Monitoring in Weight Loss: A Systematic Review of the Literature. J Am Diet Assoc.

[30] Simpson SA, McNamara R, Shaw C, Kelson M, Moriarty Y, Randell E (2015). A feasibility randomised controlled trial of a motivational interviewing-based intervention for weight loss maintenance in adults.

[31] Hutchesson MJ, Rollo ME, Krukowski R, Ells L, Harvey J, Morgan PJ (2015). eHealth interventions for the prevention and treatment of overweight and obesity in adults: a systematic review with meta-analysis. Obes Rev.

[32] Khaylis A, Yiaslas T, Bergstrom J, Gore-Felton C (2010). A Review of Efficacious Technology-Based Weight-Loss Interventions Five Key Components. Telemedicine J e-Health.

[33] Purcell R, Mcinnes S, Halcomb EJ (2014). Telemonitoring can assist in managing cardiovascular disease in primary care a systematic review of systematic reviews. BMC Fam Pract.

